# Understanding early childhood behavioral problems through parenting style–temperament typologies: a latent profile analysis

**DOI:** 10.3389/fpsyg.2025.1620464

**Published:** 2025-11-13

**Authors:** Mingyue Zhou, Dongmei Zhu, Shi Guo, Minxiao Zheng, Junhua Dang

**Affiliations:** 1 School of Education, Jianghan University, Wuhan, China; 2 School of Humanities and Social Science, Xi’an Jiaotong University, Xi’an, China; 3 Department of Surgical Sciences, Uppsala University, Uppsala, Sweden

**Keywords:** parenting style, temperament, behavioral problems, latent profile analysis, early childhood

## Abstract

**Introduction:**

This study examined the combined effects of parenting style and child temperament on early childhood behavioral problems using latent profile analysis.

**Methods:**

A total of 5,138 valid questionnaires were collected from parents of children aged 3 to 6 years in Wuhan, China. Parenting styles, temperament traits, and behavioral problems were assessed using standardized instruments.

**Results:**

Four distinct latent profiles were identified: *Dysregulated Parenting–Rebellious Temperament*, *Unstable Parenting–Reserved Temperament*, *Supportive Parenting–Challenging Temperament*, and *Enriched Parenting–Compliant Temperament*. Children in the *Dysregulated Parenting–Rebellious Temperament* group exhibited the highest rates of behavioral problems, whereas those in the *Enriched Parenting–Compliant Temperament* group showed the lowest. The results suggest that when children display more challenging temperamental traits, supportive parenting may be associated with lower behavioral problem prevalence. Conversely, children with more adaptable temperaments appear less susceptible to behavioral difficulties even in the context of less optimal parenting. Additionally, girls demonstrated significantly higher overall detection rates of behavioral problems than boys, and children with less-educated parents and lower household income exhibited greater behavioral problem prevalence.

**Discussion:**

These findings underscore the importance of promoting positive parenting practices and accounting for individual temperament differences in early prevention efforts. Future research should employ longitudinal designs to validate and extend these findings.

## 1 Introduction

Behavioral problems in early childhood can lead to significant impairments in peer relationships, delays in social skill acquisition, academic underachievement, and other developmental challenges, which may in turn contribute to negative long-term outcomes (Parker et al., 2021). Longitudinal research has shown that early behavioral difficulties are associated with a range of adverse consequences in adulthood, including early school dropout, lower educational attainment, unemployment, early parenthood, relationship instability (e.g., divorce or separation), substance abuse, and increased risk of psychiatric disorders such as depression and anxiety (Colman et al., 2009).

Temperament, as an innate psychological characteristic of individuals, forms the cornerstone of behavioral development in young children. Temperament type is closely linked to psychological and behavioral problems during early childhood (Zou et al., 2010). For example, impulsivity is negatively associated with internalizing problems, whereas fear is positively associated with internalizing problems (Karreman et al., 2010). Children with inhibited temperaments are more likely to exhibit externalizing behavior problems in middle and late childhood (Song et al., 2016). Moreover, children with different temperament types display distinct emotion regulation strategies when faced with external stimuli, leading to varying behavioral traits. For instance, Liu et al. (2019) found that young children scoring higher on the positive affective dimension of temperament tended to show more impulsive behaviors, those with higher anger scores exhibited poorer attentional control and greater impulsivity, and those with higher behavioral inhibition scores were more prone to anxiety symptoms, particularly social anxiety.

While temperament is innate and plays a significant role in psycho-behavioral development, parenting style—a key environmental factor—also critically influences behavioral outcomes in young children (Alizadeh et al., 2011; Hoeve et al., 2009; Hosokawa and Katsura, 2019; Sumargi et al., 2020). For example, Braza et al. (2015) found that authoritarian maternal styles were positively associated with internalizing problems in young children. Moreover, the combination of authoritarian mothering and permissive fathering was negatively associated with internalizing problems in boys but positively associated with aggressive behavior in both boys and girls. Permissive parenting was positively related to physical aggression in girls, though no significant association was found in boys. Furthermore, appropriate parenting styles can mitigate behavioral problems and promote healthy child development (Liang et al., 2024).

Traditional classifications of parenting styles—authoritative, democratic, and permissive—primarily emphasize observable behavioral patterns. However, real-world parenting often involves a mixture of these styles. Darling and Steinberg (1993) proposed a more comprehensive model that encompasses three dimensions: parenting beliefs, parenting emotions, and parenting behaviors. Parenting beliefs shape how parents interpret and respond to their children’s behavior; parenting emotions reflect the degree of emotional support and care; and parenting behaviors capture the specific ways parents interact with their children. Based on this framework, the present study analyzes parenting style across these three dimensions.

There is a longstanding tradition of theoretical and empirical research on the structure of child temperament, beginning with Thomas and Chess’s (1977) typology of easy, slow-to-warm-up, and difficult children. However, most earlier studies have employed variable-centered approaches, which aggregate data across individuals and may obscure meaningful person-level heterogeneity (Fisher et al., 2018). In contrast, recent advances in person-centered methods—particularly latent profile analysis (LPA)—have enabled researchers to uncover distinct temperament profiles that reflect natural combinations of traits within individuals. For example, Rettew et al. (2008) used LPA to identify three temperament types among children: a moderate class, a steady class (low novelty seeking, high persistence), and a disengaged class (high novelty seeking and harm avoidance, low reward dependence and persistence). Similarly, Gartstein et al. (2017) found that the optimal number of temperament profiles varied across developmental stages in infancy, with a three-class solution emerging for infants aged 3–8 months and a five-class solution for those aged 9–12 months, underscoring the dynamic nature of temperament. In parallel with the application of LPA in temperament research, recent studies have also begun to use person-centered approaches to classify parenting styles. For example, Wu et al. (2016) identified three parenting types—positive, negative, and mixed—based on adolescents’ perceptions, while Zhang and Xiao (2023) extracted positive, inconsistent, and negative parenting profiles among secondary school students. These findings demonstrate that LPA offers a more holistic and statistically robust means of capturing the complex, multidimensional nature of parenting. Compared to variable-centered approaches, which treat temperament traits as independent predictors, person-centered methods like LPA identify meaningful configurations of traits that may better explain developmental outcomes (Hawes et al., 2022).

Decades of developmental theory have emphasized that a child’s outcomes depend not only on individual factors but on the fit between the child and their rearing environment (Gartstein et al., 2024; Zubizarreta et al., 2019). Classic Goodness-of-Fit theory posits that optimal development arises when parenting behaviors are well matched to a child’s temperament. Thomas and Chess (1977) originally used this framework to explain why children with “difficult” temperaments fare better with caregiver strategies tailored to their style. This perspective marked a shift from viewing parenting as a one-directional influence to recognizing bidirectional dynamics: children’s temperamental characteristics can alter how parents respond (Liu et al., 2023). In other words, what is “good” parenting may depend on who the child is—a notion that underlies modern person–environment interaction models (Xia et al., 2017).

More recent theories elaborate on why specific parenting–temperament combinations matter. The Diathesis–Stress model (also called “dual risk”) proposes that children with certain vulnerabilities (e.g., high negative emotionality) are disproportionately affected by adverse parenting, leading to elevated risk for problems under stress, though they show typical outcomes under nurturing conditions (Walker et al., 1989). Differential Susceptibility theory extends this idea: it argues that some children are not only more vulnerable to negative environments but also benefit more than others from positive, supportive parenting (Belsky and Pluess, 2009). In this framework, a highly reactive or “orchid” child might thrive exceptionally well with sensitive parenting but struggle markedly with harsh discipline. Both differential susceptibility and diathesis–stress models build on the Goodness-of-Fit principle by explaining why children with similar temperaments can have divergent outcomes in the same parenting context. They highlight that parenting style and child temperament jointly shape development, aligning with the view that neither factor is uniformly “good” or “bad” in isolation—it’s the combination that matters.

A wealth of empirical evidence from variable-centered studies supports this bidirectional interaction between temperament and parenting. Numerous studies have found that the effect of parenting practices on child behavior is moderated by child temperament, and vice versa. For example, a meta-analytic review by Kiff et al. (2011) concluded that ineffective or harsh parenting predicts worse mental health primarily among children with vulnerable temperaments: coercive discipline was associated with heightened behavior problems especially in highly irritable (easily angered) children, and inconsistent discipline or low warmth predicted greater internalizing symptoms in children low in self-regulation. Similarly, parental overprotection and intrusiveness have been linked to anxiety mostly for temperamentally fearful kids. These patterns echo Thomas and Chess’s (1977) original insight—a poor fit between parenting and temperament can precipitate maladjustment. Conversely, evidence for differential susceptibility has also emerged: in one longitudinal study, children high in negative emotionality exhibited more externalizing problems than their peers under harsh, punitive parenting, but fewer problems than others when raised with high warmth and sensitivity (Loginova and Slobodskaya, 2021). Such findings illustrate that children respond to parenting in individualized ways. The parent–child dynamic is transactional, with each influencing the other over time, which reinforces the idea that we must consider them in tandem.

Given this theoretical and empirical backdrop, it is logical to combine parenting style and child temperament into latent profiles. A person-centered approach like LPA allows us to identify subgroups of individuals characterized by particular parenting–temperament constellations, rather than examining each variable in isolation. This approach directly captures the nuanced interplay (or “goodness of fit”) that theories describe. By clustering families into latent profiles (for instance, “highly reactive child with very permissive parent” vs. “easygoing child with structured, warm parent”), we can test whether certain profiles yield different child outcomes, aligning with the idea that some parent–child matches are more adaptive than others. Recent research has begun to adopt this strategy. For example, a latent profile study of infants in a Korean context identified distinct temperament–parenting profiles such as “Low Regulation–Low Support” versus “Well-Regulated–High Support” (Kim, 2025). These profiles were differentially associated with developmental outcomes, with the mismatched “Low Regulation–Low Support” infants showing the poorest competence. Such person-centered findings underscore the importance of a developmentally attuned match between child temperament and parenting behavior. In sum, forming latent profiles that integrate parenting style and temperament is not only theoretically justified by Goodness-of-Fit and susceptibility models, but it is also supported by prior evidence that the synergy between a child’s temperament and the parenting they receive is predictive of behavioral outcomes beyond the sum of their parts.

Therefore, this study treats temperament and parenting style as two interrelated yet distinct factors and aims to examine their joint influence on young children’s behavioral problems using LPA. As a person-centered approach, LPA allows for the identification of heterogeneous subgroups within the population, offering a more nuanced understanding of how combinations of parenting and temperament characteristics relate to developmental outcomes (Su et al., 2015). Specifically, this study hypothesizes that: (1) Parenting styles and child temperament can each be categorized into distinct latent classes; and (2) Behavioral problems will vary significantly across these combined classes, with evidence of compensatory effects—such that positive parenting may buffer the negative impact of difficult temperament, and conversely, favorable temperament traits may mitigate the risks associated with suboptimal parenting.

To better interpret the associations between parenting styles, temperament, and behavioral problems, it is necessary to first examine how these outcomes may differ across basic demographic variables. Prior research has shown that behavioral problems in early childhood are associated with factors such as gender, family structure, parental education, and socioeconomic status (Dubow et al., 2009; Fu, 2023; Owens, 2016). These demographic variables not only provide essential context for understanding the distribution of behavioral problems but also help clarify whether observed differences across latent profiles are attributable to parenting and temperament factors, or are confounded by background characteristics. Therefore, we first conducted descriptive and group comparison analyses to explore the influence of key demographic variables—including gender, family type, parental education, and income—on children’s behavioral problem scores. These analyses support the interpretation of the LPA-derived profiles and provide additional insight into how demographic risk factors may independently relate to behavioral outcomes.

## 2 Methods

### 2.1 Study setting

This study was conducted in Wuhan City, Hubei Province, China, using a cluster random sampling method across eight urban districts. Data were collected during scheduled kindergarten sessions in 30 participating schools, where caregivers uniformly completed electronic surveys by scanning QR codes distributed through the Questionnaire Star platform, under supervised conditions. The study protocol was approved by the Academic Ethics Committee of the Maternal and Child Health Hospital of East–West Lake District, Wuhan (Approval No. 20230322012).

### 2.2 Participants

The final sample comprised 5,138 valid parents responses (95.77% response rate) representing children aged 3–6 years (*M* = 5.45, SD = 0.89). The sample included 2,653 boys (51.60%) and 2,485 girls (48.40%), with all participants meeting the inclusion criteria of being typically developing children attending regular kindergarten programs. Both classroom teachers and parents provided informed consent prior to participation, ensuring adherence to ethical guidelines throughout the study.

### 2.3 Variables and measurements

#### 2.3.1 Demographic information

Demographic information was collected using a self-administered questionnaire, including the child’s sex, age, family type, parental education level and income. Family types were categorized as: nuclear family (parents and minor children), extended family (three generations living together), single-parent or reorganized family, and others. Parental education levels were classified as junior high school or below, senior high school (including vocational school), university (including college), and master’s degree or above. Parental annual income was categorized into four groups: less than 50,000 RMB, 60,000–100,000 RMB, 100,000–150,000 RMB, and above 150,000 RMB.

#### 2.3.2 Parenting styles

Parenting styles were assessed using the revised version of the Parental Bonding Instrument (PBI) developed by Li (2022), which includes 22 items covering three dimensions: parenting emotions, parenting beliefs, and parenting behaviors. Each item was rated on a 4-point Likert scale (0 = very inconsistent, 1 = relatively inconsistent, 2 = relatively consistent, 3 = very consistent). In the current study, the scale demonstrated excellent internal consistency, with a Cronbach’s alpha coefficient of 0.92. Example items include: “You speak to your child in a warm and friendly tone” and “You do not provide your child with sufficient assistance.”

#### 2.3.3 Temperament

Temperament was measured using the Parent Temperament Questionnaire (PTQ) developed by Thomas and Chess (1977), consisting of 72 items covering nine dimensions: activity level, rhythmicity, avoidance, adaptability, response intensity, emotional nature, persistence, attentional distraction, and response threshold. Items were rated on a 7-point scale (1 = never happens, 7 = always happens). The Cronbach’s alpha coefficient for this scale in the current study was 0.89. Example items include: “During bath time, splashes water around and plays vigorously” and “Appears joyful when playing with other children.”

#### 2.3.4 Behavioral problems

Behavioral problems were assessed using the Behavioral Problems subscale of the Child Behavior Checklist (CBCL) for ages 4–16, compiled by American psychologist Achenbach and adapted by Huang et al. (2022). The subscale contains 113 items covering eight behavioral symptom factors: Withdrawal, Somatic Complaints, Anxiety/Depression, Social Problems, Thinking Problems, Attention Problems, Rule-Breaking Behavior, and Aggressive Behavior. Each item was rated on a 3-point scale (0 = not true, 1 = somewhat or sometimes true, 2 = very true or often true). Total scores were obtained by summing up the raw scores of all relevant items. In this study, the Cronbach’s alpha coefficient for the subscale was 0.96, indicating excellent reliability. In addition, detection rates were calculated based on whether a child’s score in a given dimension or the total behavioral problems score exceeded the 98th percentile; scores above this threshold were considered indicative of behavioral abnormalities. Example items include: “Refuses to eat properly” and “Does not interact with other children.”

### 2.4 Statistical analysis

All scale items were analyzed using standardized scores. LPA was conducted in Mplus 8.7, with optimal model selection determined primarily by the Akaike Information Criterion (AIC), Bayesian Information Criterion (BIC), adjusted Bayesian Information Criterion (aBIC), entropy, Lo–Mendell–Rubin Adjusted LRT Test (LMR), and Bootstrap Likelihood Ratio Test (BLRT). Lower values of AIC, BIC, and aBIC indicate better model fit. Entropy ranges from 0 to 1, with values closer to 1 denoting more precise classification. Significant LMR and BLRT tests (*p* < 0.05) indicate that the k-class model fits significantly better than the (k-1)-class model. The number of classes was progressively increased from the initial model until the best-fitting solution was identified. For group comparisons based on the retained optimal class solution, chi-square tests (χ^2^) were performed in SPSS 27.0, with statistical significance set at *p* < 0.05 (two-tailed).

## 3 Results

### 3.1 Detection of behavioral problems in young children

As shown in Tables 1, 2, the detection rate of boys was higher than that of girls in the dimensions of anxiety/depression (*p* = 0.010) and aggressive behavior (*p* = 0.042). Conversely, girls showed significantly higher detection rates than boys in the dimensions of social problems, attention problems, rule-breaking behaviors, and in the total score of behavioral problems (*p*s < 0.001). There was no significant difference in behavioral problems based on family type (*p* = 0.101). However, significant differences were observed based on parental education level and total annual household income (*p*s < 0.001). Specifically, children with less-educated parents and lower annual household income demonstrated significantly higher detection rates of behavioral problems (Table 3).

**Table 1:** Detection of behavioral problems in children of different genders [n (%)].

Factor	Male		Female		*χ* ^2^	*p*
	Normalcy	Abnormality	Normalcy	Abnormality		
Withdrawal	2,568 (96.8)	85 (3.2)	2,393 (96.3)	92 (3.7)	0.958	0.328
Somatic complaint	2,617 (98.6)	36 (1.4)	2,436 (98.0)	49 (2.0)	2.982	0.084
Anxiety/depression	2,528 (95.3)	125 (4.7)	2,403 (96.7)	82 (3.3)	6.615	0.010
Social problem	2,387 (99.0)	266 (1.0)	2,382 (95.9)	103 (4.1)	66.586	0.000
Thinking problem	2,630 (99.1)	23 (0.9)	2,468 (99.3)	17 (0.7)	0.555	0.456
Attention problem	2,588 (97.5)	65 (2.5)	2,364 (95.1)	121 (4.9)	21.523	0.000
Rule-breaking	2,630 (99.1)	23 (0.9)	2,407 (96.9)	78 (3.1)	34.367	0.000
Aggressive behavior	2,487 (93.7)	166 (6.3)	2,362 (95.1)	123 (4.9)	4.131	0.042
Total score	2,455 (92.5)	198 (7.5)	2,230 (89.7)	255 (10.3)	12.498	0.000

**Table 2:** Analysis of variance in the prevalence of behavioral problems across demographic variables [*n*(%)].

Variant	Event	Number	Abnormality	*χ* ^2^	*p*
Type of family	Extended family	2,986	283 (9.5)	6.218	0.101
	Nuclear family	1,943	150 (7.7)		
	Single parent or reorganized family	89	6 (6.7)		
	Other	120	14 (11.7)		
Father’s educational level	Junior high school and below	614	86 (14.0)	36.968	0.000
	High school (including secondary school)	1,251	128 (10.2)		
	University (including college)	2,933	224 (7.6)		
	Master’s degree or above	340	15 (4.4)		
Mother’s educational level	Junior high school and below	662	93 (14.0)	54.264	0.000
	High school (including secondary school)	1,183	138 (11.7)		
	University (including college)	3,017	210 (7.0)		
	Master’s degree or above	276	12 (4.3)		
Father’s annual income	<50,000	612	76 (12.4)	28.018	0.000
	600,000–100,000	1,599	169 (10.6)		
	100,000–150,000	1,337	104 (7.8)		
	>150,000	1,590	104 (6.5)		
Mother’s annual income	<50,000	2,136	249 (11.7)	41.126	0.000
	600,000–100,000	1,571	123 (7.8)		
	100,000–150,000	845	49 (5.8)		
	>150,000	586	32 (5.5)		

**Table 3:** Correlation analysis between temperament and parenting styles.

	1	2	3	4	5	6	7	8	9	10	11
1. Parenting concepts	–										
2. Parenting emotions	0.642**	–									
3. Parenting behaviors	0.507**	0.619**	–								
4. Activity level	−0.181**	−0.235**	−0.204**	–							
5. Response intensity	−0.094**	−0.154**	−0.128**	0.304**	–						
6. Response threshold	−0.293**	−0.301**	−0.269**	0.189**	0.027**	–					
7. Persistence	−0.025	−0.037**	−0.010	0.504**	−0.207**	0.055**	–				
8. Rhythmicity	0.229**	0.284**	0.206**	−0.242**	−0.128**	−0.318**	0.012	–			
9. Avoidance	0.225**	0.255**	0.278**	−0.104**	−0.218**	0.276**	−0.019**	0.197**	–		
10. Adaptability	0.336**	0.353**	0.370**	−0.219**	−0.271**	−0.378**	−0.158**	0.292**	0.598**	–	
11. Emotional nature	0.334**	0.390**	0.356**	−0.221**	−0.294**	−0.336**	−0.091**	0.329**	0.379**	0.514**	–
12. Attentional distraction	0.280**	0.319**	0.268**	−0.178**	−0.263	−0.285**	−0.266**	0.207**	0.331**	0.526**	0.417**

*p < 0.05, **p < 0.01, ***p < 0.001.

### 3.2 Analysis of potential categories of parenting style-temperament

Following the method of Ma and Yang (2024), this study calculated and summarized the fit indices for five models to estimate and compare model fit in LPA while allowing item variances to vary across classes. The fit evaluation criteria included the Akaike Information Criterion (AIC), Bayesian Information Criterion (BIC), adjusted BIC (aBIC), Entropy, and the Lo–Mendell–Rubin likelihood ratio test (LMR) and Bootstrap Likelihood Ratio Test (BLRT). Specifically, lower values of AIC, BIC, and aBIC and an Entropy value closer to 1 indicate better model fit (Wang et al., 2017). Additionally, a *p*-value less than 0.05 for the LMR and BLRT tests suggests a significantly better model fit (Carragher et al., 2009).

As shown in Table 4, model fit indices (e.g., AIC, BIC) consistently improved with the addition of more classes. However, the rate of improvement diminished markedly after the 4-class model. Although the 2-class and 3-class solutions yielded slightly higher entropy values than the 4-class model, entropy alone should not determine model selection (Masyn, 2013; Nylund et al., 2007). While entropy reflects classification precision, it does not necessarily indicate the psychological interpretability or practical utility of the profiles. Although the 5-class model demonstrated marginally lower AIC and BIC values than the 4-class solution, it introduced a class representing only 5.5% of the sample. This small subgroup lacked clear psychological distinctiveness and may reflect statistical noise rather than a meaningful latent class. Prior LPA research has cautioned against over-extraction of classes, particularly when additional profiles comprise fewer than 5% of the sample and lack theoretical coherence (Masyn, 2013). Such small classes are typically unstable and less likely to replicate across independent samples.

**Table 4:** Summary of potential profile analysis fitting information (*N* = 5,138).

Models	AIC	BIC	aBIC	Entropy	LMR(*p*)	BLRT(*p*)	Class proportions	Average posterior probability
1	175,008.136	175,165.202	175,088.938	–	–	–	–	–
2	164,464.113	164,784.789	164,629.084	0.799	0.000	0.000	0.531/0.469	0.933/0.947
3	161,001.257	161,485.544	161,250.397	0.830	0.000	0.000	0.137/0.305/0.559	0.926/0.913/0.922
4	159,463.867	160,033.232	159,756.775	0.782	0.000	0.000	0.121/0.247/0.296/0.336	0.916/0.891/0.880/0.842
5	158,105.933	158,760.375	158,442.609	0.782	0.040	0.041	0.227/0.055/0.199/0.229/0.291	0.903/0.934/0.870/0.826/0.820
6	157,398.311	158,137.830	157,778.754	0.781	0.004	0.004	0.220/0.032/0.245/0.190/0.072/0.241	0.823/0.961/0.812/0.888/0.869/0.824

**Figure 1:**
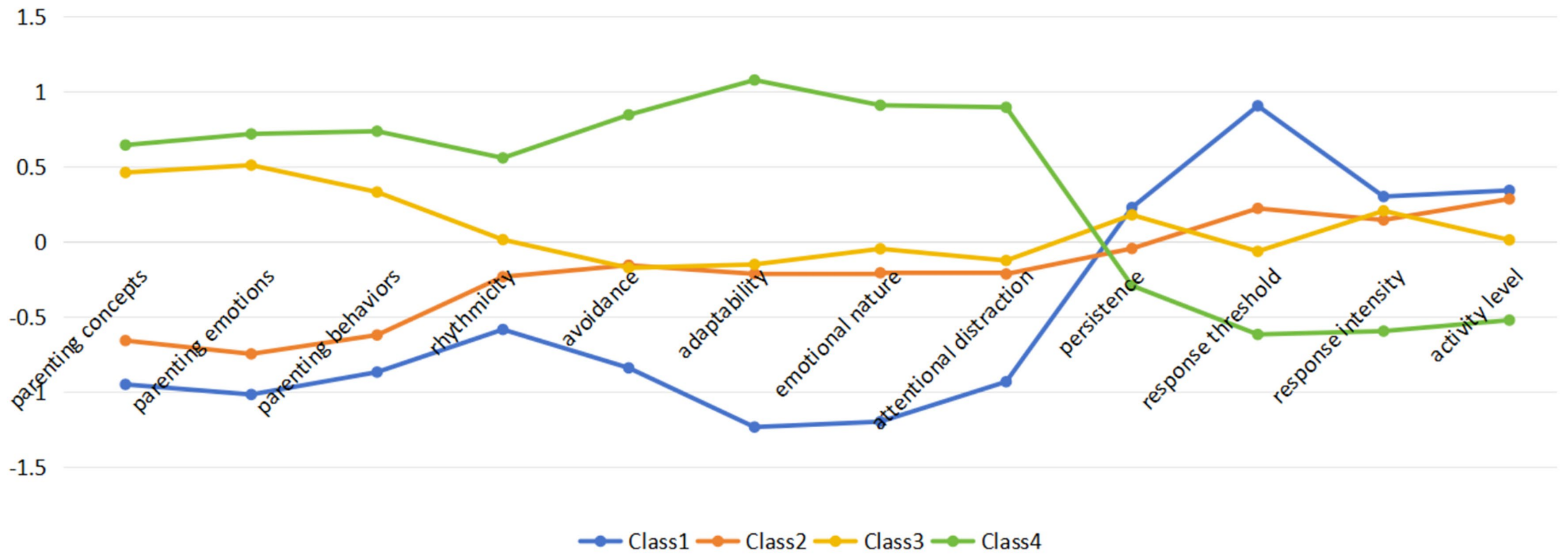
The means of the indicator variables for each class.

In contrast, the 4-class solution showed balanced class sizes (ranging from 12.1 to 33.6%) and acceptable entropy (0.782), with posterior classification probabilities exceeding 0.842 for all classes, suggesting reliable profile separation. Most importantly, the four-profile solution provided psychologically coherent and theoretically meaningful combinations of parenting style and temperament traits. These configurations aligned well with established frameworks such as the goodness-of-fit model (Thomas and Chess, 1977) and differential susceptibility theory (Belsky and Pluess, 2009), which emphasize the dynamic interaction between child characteristics and environmental influences. Notably, the four profiles encompassed all key combinations of parenting and temperament—supportive vs. dysregulated parenting crossed with easy vs. difficult temperament—thus capturing both compensatory and risk-amplifying patterns. For example, as shown in Figure 1, Class 4 reflects an optimal pairing of responsive caregiving and emotionally adaptable children, while Class 1 reflects the convergence of two vulnerabilities. The remaining two profiles illustrate intermediate, compensatory dynamics: children with difficult temperaments buffered by supportive parenting (Class 3), and children with relatively easy temperaments exposed to suboptimal caregiving (Class 2). This balanced representation of interaction types offers a richer framework for understanding the multifaceted nature of early behavioral development. Therefore, based on a combination of statistical fit, classification precision, conceptual interpretability, and model parsimony, we selected the 4-class model as the final solution. In supplementary materials, we also provided figures and tables displaying the class-specific means for all indicator variables across the 2- to 5-class models.

The responses of the four latent categories regarding family parenting style and temperament are shown in Figure 1. Based on the parenting style scores, the four categories were named “Dysregulated Parenting,” “Unstable Parenting,” “Supportive Parenting,” and “Enriched Parenting.” Regarding temperament dimensions, the first category was characterized by the lowest scores in regularity, avoidance, adaptability, and emotional nature, and the highest score in response threshold; therefore, it was labeled “Rebellious Temperament.” The second category showed lower scores in activity level, response intensity, and regularity, along with a higher score in emotional nature, and was named “Reserved Temperament.” The third category had lower scores in regularity, avoidance, adaptability, and emotional nature, and a higher score in response intensity, leading to the name “Challenging Temperament.” The fourth category exhibited higher scores in regularity, avoidance, adaptability, and emotional nature, and lower response intensity, and was therefore named “Compliant Temperament.” Combining the results of parenting style and temperament, the four latent profiles were ultimately labeled as “Dysregulated Parenting–Rebellious Temperament,” “Unstable Parenting–Reserved Temperament,” “Supportive Parenting–Challenging Temperament,” and “Enriched Parenting–Compliant Temperament.” Among them, the Dysregulated Parenting–Rebellious Temperament” group included 622 participants (12.1%), the “Unstable Parenting–Reserved Temperament” group included 1,269 participants (24.7%), the “Supportive Parenting–Challenging Temperament” group included 1,521 participants (29.6%), and the “Enriched Parenting–Compliant Temperament” group included 1,726 participants (33.6%).

### 3.3 Differences in early childhood behavioral problems across latent profiles

As shown in Table 5, the “Dysregulated Parenting–Rebellious Temperament” group had the highest detection rates across all behavioral problem dimensions and the total behavioral problems score, while the “Enriched Parenting–Compliant Temperament” group had the lowest detection rates. The detection rates for the “Unstable Parenting–Reserved Temperament” and “Supportive Parenting–Challenging Temperament” groups fell in between. Pairwise comparisons between the latent profile groups revealed significant differences in all dimensions of behavioral problems and in the total behavioral problems score between the “Dysregulated Parenting–Rebellious Temperament” group and each of the other three groups (*p* < 0.001). Additionally, a significant difference was observed in the withdrawal dimension between the “Unstable Parenting–Reserved Temperament” and “Supportive Parenting–Challenging Temperament” groups (*p* < 0.01), with the detection rate for withdrawal being higher in the “Supportive Parenting–Challenging Temperament” group. Furthermore, the “Enriched Parenting–Compliant Temperament” group differed significantly from both the “Unstable Parenting–Reserved Temperament” and “Supportive Parenting–Challenging Temperament” groups across all dimensions of behavioral problems and the total behavioral problems score (*p* < 0.001).

**Table 5:** Comparison of the detection rates of children’s behavioral problems in each latent profile.

Factor	Class 1 (*N* = 1,064)	Class 2 (*N* = 1,481)	Class 3 (*N* = 1,395)	Class 4 (*N* = 1,198)	*χ* ^2^
Withdrawal	114 (10.7)	19 (1.3)	41 (2.9)	3 (0.3)	227.676 ***
Somatic complaint	59 (5.5)	12 (0.8)	12 (0.9)	2 (0.2)	127.183 ***
Anxiety/depression	121 (11.4)	34 (2.3)	41 (2.9)	11 (0.9)	194.163 ***
Social problem	181 (17.0)	88 (5.9)	79 (5.7)	21 (1.8)	215.429 ***
Thinking problem	66 (6.2)	33 (2.2)	39 (2.8)	14 (1.2)	55.264 ***
Attention problem	104 (9.8)	46 (3.1)	29 (2.1)	7 (0.6)	157.768 ***
Rule-breaking	64 (6.0)	19 (1.3)	17 (1.2)	1 (0.1)	120.179 ***
Aggressive behavior	142 (13.3)	80 (5.4)	59 (4.2)	8 (0.7)	180.201 ***
Total score	211 (19.8)	125 (8.4)	100 (7.2)	17 (1.4)	247.080 ***

*p < 0.05, **p < 0.01, ***p < 0.001.

## 4 Discussion

This study identified four distinct parenting-temperament profiles among Chinese preschoolers. Key findings show that positive parenting can buffer behavioral risks even for challenging temperaments (“Enriched Parenting–Compliant Temperament” group had lowest problem rates), while difficult temperaments combined with Dysregulated Parenting yielded worst outcomes. Girls showed higher behavioral problems than boys, and lower parental SES predicted increased risks. These results highlight the importance of tailored interventions considering both parenting quality and child temperament.

Specifically, it was found that the overall detection rate of behavioral problems was higher in girls than in boys. This may be attributed to the fact that the survey relied on parental reporting, and parents may have been more sensitive to and critical of behaviors in girls that deviated from traditional gender roles, thereby leading to a higher reported detection rate of behavioral problems among girls. Specifically, boys exhibited higher detection rates for anxiety/depression and aggressive behavior, while girls showed higher detection rates for social problems, attention problems, and disciplinary behaviors. These differences may stem from the gender role socialization process during early childhood. Boys are often encouraged to display traits such as bravery, competitiveness, and aggression, whereas girls are taught to be friendly, cooperative, and submissive. This differential socialization may lead boys to express emotional distress through externalized behaviors, such as aggression, while girls are more likely to internalize emotional issues, manifesting through social, attention, and disciplinary problems rather than overt aggression. Furthermore, this study further revealed that young children with less-educated parents and lower household incomes exhibited higher detection rates of behavioral problems, aligning with prior findings (Reiss, 2013). According to the family investment model, parents of higher socioeconomic status are equipped with better economic, social, and human capital and are more likely to invest substantially in their children’s development, which may indirectly reduce the incidence of behavioral problems.

This study employed a person-centered approach to jointly examine parenting styles and child temperament, identifying four distinct combined profiles. The first category, “Dysregulated Parenting–Rebellious Temperament,” comprised children whose parents demonstrated maladaptive parenting beliefs, including limited awareness of developmental needs and inadequate responsiveness to children’s emotional signals. Emotional expressions within the parent–child relationship were marked by negativity or emotional withdrawal, with minimal warmth or affective attunement. Parenting behaviors fluctuated between inconsistent control and permissive disengagement, reflecting a lack of structure and reliability. Children in this group showed strong internal arousal (e.g., high activity level) but had marked difficulties with environmental regulation, such as irregular daily routines and low adaptability to novel situations. They often displayed oppositional tendencies and interpersonal conflict. The second category, “Unstable Parenting–Reserved Temperament,” reflected a slightly more functional parenting pattern than the first group. Parents exhibited moderate emotional warmth and partial consistency in behavior, though still lacked coherence in belief and behavior alignment. Children in this group demonstrated relatively adequate external adaptation but exhibited vulnerabilities in emotional self-regulation—for example, prolonged crying in response to minor setbacks—suggesting less mature coping strategies. The third category, “Supportive Parenting–Challenging Temperament,” featured parents who were generally attuned to their children’s developmental and emotional needs. They engaged in warm, responsive interactions, using gentle communication, positive reinforcement, and supportive guidance while fostering autonomy through age-appropriate choice and involvement. Children in this group exhibited temperament traits similar to those in the first category—such as elevated activity and persistence—but with fewer difficulties in adapting to environmental changes, possibly due to the buffering role of consistent and supportive caregiving. The fourth category, “Enriched Parenting–Compliant Temperament,” represented the most adaptive profile. Parents in this group displayed high levels of warmth, consistent behavioral routines, and a nuanced understanding of child development, fostering both structure and emotional security. Their children, while somewhat lower in internal activation (e.g., moderate activity or persistence), showed strong environmental adaptability, including regular sleep–wake patterns, openness to novelty, emotional stability, and flexible adjustment in social situations.

Children in the Dysregulated Parenting–Rebellious Temperament group exhibited the most pronounced behavioral difficulties. Parenting characterized by inconsistency, low emotional responsiveness, and oscillation between control and neglect has been shown to disrupt the quality of the parent–child relationship, increase relational conflict, and elevate the risk for behavioral problems (Ma et al., 2021). When such parenting is combined with a temperament marked by high reactivity, low adaptability, and poor regulation, the likelihood of behavioral disturbances is further exacerbated (Muhtadie et al., 2013). In contrast, children in the Enriched Parenting–Compliant Temperament group displayed the lowest incidence of behavioral problems, highlighting the protective effect of responsive, structured parenting combined with an emotionally stable and adaptable temperament. This profile appears most conducive to healthy developmental outcomes.

Behavioral problems in the Unstable Parenting–Reserved Temperament and Supportive Parenting–Challenging Temperament groups were moderate in severity. This suggests that when either the caregiving environment or the child’s temperament is favorable, it can buffer the impact of the other, mitigating the overall risk for behavioral difficulties. Notably, these two groups differed in the withdrawal dimension: children in the Supportive Parenting–Challenging Temperament group showed higher levels of withdrawal than those in the Unstable Parenting–Reserved Temperament group. One possible explanation is that although the parenting in the former group was more consistent and warm, the children’s challenging temperament—characterized by emotional sensitivity and lower adaptability—rendered them more susceptible to withdrawal in response to frustration or perceived failure. In contrast, children with a reserved temperament, though exposed to less optimal parenting, may possess greater internal stability and adaptive capacity, which may help buffer the negative effects of the caregiving environment. Crucially, despite the presence of more difficult temperamental traits, children who received Supportive Parenting still had significantly fewer behavioral problems across all dimensions compared to those in the Dysregulated Parenting–Rebellious Temperament group. This finding underscores the moderating role of parenting in shaping behavioral outcomes and supports the view that positive caregiving practices can offset the risks posed by less adaptive temperaments (Li et al., 2012).

These findings not only reinforce prior research but also contribute new insights to the literature on parenting and temperament. While some components of the identified profiles align with existing classifications—for instance, the Compliant Temperament in our study resembles the “Steady Class” described by Rettew et al. (2008), and the Dysregulated Parenting pattern echoes Zhang and Xiao’s (2023) findings on negative parenting—our results provide two key extensions. First, by jointly modeling parenting style and temperament, we identified systematic co-occurrence patterns, such as the association between emotionally inconsistent, overcontrolling parenting and children’s high negative reactivity and low adaptability. Conversely, we observed that supportive parenting could also emerge in the context of challenging temperaments, such as high impulsivity or elevated activity levels, suggesting that some parents adapt constructively to temperamental difficulty. Second, these findings challenge the assumptions of coercive cycle theory (Patterson, 1982), which proposes that difficult temperament tends to evoke increasingly harsh parenting. Our results indicate that this dynamic is not inevitable: when parents respond with warmth, consistency, and emotional attunement, they may buffer, mitigate, or even reshape the expression of difficult temperament traits in young children.

Nevertheless, several limitations should be noted. First, behavioral problem data were obtained exclusively through parental reports, which may be subject to bias due to social desirability or perceptual inaccuracy. Second, the cross-sectional design precludes causal inference and limits understanding of how parenting styles and temperament interact to shape behavioral development over time. Third, the study did not differentiate between maternal and paternal parenting styles, instead aggregating them into a single construct. This approach may have masked important differences in how mothers and fathers individually contribute to children’s behavioral outcomes. Future research should employ longitudinal designs to assess developmental trajectories and examine how changes in parenting and temperament interact over time. In addition, disaggregating maternal and paternal influences could provide a more nuanced understanding of parental roles in early childhood behavioral adjustment.

## 5 Conclusion

This study examined the joint influence of parenting style and child temperament on early childhood behavioral problems using a person-centered latent profile analysis approach. Four distinct parenting style–temperament profiles were identified: Dysregulated Parenting–Rebellious Temperament, Unstable Parenting–Reserved Temperament, Supportive Parenting–Challenging Temperament, and Enriched Parenting–Compliant Temperament. Children in the Dysregulated Parenting–Rebellious Temperament group exhibited the highest levels of behavioral problems, while those in the Enriched Parenting–Compliant Temperament group showed the lowest. The findings indicate that a supportive parenting environment can buffer the adverse effects of difficult temperamental traits, and conversely, that children with more adaptable temperaments may show resilience even when parenting practices are suboptimal. These results underscore the importance of designing intervention strategies that are responsive not only to general parenting practices but also to children’s individual temperament characteristics. Moreover, given the observed gender differences in behavioral problem profiles, gender-sensitive interventions are warranted. For instance, boys may benefit from programs that enhance emotional awareness, reduce impulsivity, and redirect aggressive behaviors into constructive outlets, while girls may require support aimed at improving attention regulation, social confidence, and assertiveness in peer interactions. Parenting education programs should also address gender-based biases in caregiving, encouraging more equitable and developmentally attuned responses to behavioral expressions across genders. Future longitudinal research is needed to trace the dynamic interplay between parenting and temperament over time and to evaluate the long-term effectiveness of differentiated parenting interventions. Such work will contribute to a more comprehensive understanding of how individualized, gender-informed approaches can promote healthy behavioral development in early childhood.

## Data Availability

The raw data supporting the conclusions of this article will be made available by the authors without undue reservation.
